# Coping and Protective Factors of Mental Health: An Examination of African American and US Caribbean Black Women Exposed to IPV from a Nationally Representative Sample

**DOI:** 10.3390/ijerph192215343

**Published:** 2022-11-20

**Authors:** Regina N. Parnell, Krim K. Lacey, Maxine Wood

**Affiliations:** 1 Department of Occupational Therapy, Wayne State University, Detroit, MI 48201, USA; 2 Department of Sociology and African and African American Studies, University of Michigan-Dearborn, Dearborn, MI 48128, USA; 3 Department of Humanities, York University, Toronto, ON M3J 1P3, Canada

**Keywords:** religiosity, spirituality, social support, resilience, mental health, IPV

## Abstract

Existing research continues to highlight the harm that intimate partner violence (IPV) can pose to health and well-being. However, little is done to understand the effectiveness of coping and protective mechanisms in helping women manage under adverse circumstances. The current study addresses the mental health of US Black women and the role of coping and protective moderators. An analysis of data from the National Survey of American Life (2001–2003), the most comprehensive survey on the health of US Blacks, was conducted. The association between severe physical intimate partner violence and mental health outcomes were confirmed. Resilience moderated the relationship between severe intimate partner violence and mood disorder among US Black women, but this differed between African American and Caribbean Blacks. Resilience, emotional family support, and spirituality reduced the likelihood of having a mental health condition for some African American and Caribbean Black women, while the opposite was found for religiosity. Demographic factors were also associated with mental health conditions and behaviors. The study draws our attention to potential coping and protective mechanisms that could be incorporated into counseling and intervention practices while recognizing factors that may be harmful to the mental health of individuals.

## 1. Introduction

It is well documented that victims of intimate partner violence (IPV) are vulnerable to various mental health risks and adverse outcomes [1,2,3,4,5,6]. Women of color and immigrant women are likely to endure these outcomes as they experience IPV at a rate that is higher than other populations [7,8]. It is estimated that 45.1% of US Black women, including Caribbean Black women, were victims of physical intimate partner violence [9]. While research has shown varied mechanisms through which women cope and deal with intimate victimization [10], less is known about the effectiveness of these protective factors. A better understanding might be instrumental for the development of intervention and coping strategies.

Individual attributes and social support can potentially moderate mental health risks [11,12]. Racial and ethnic minorities have been known to employ various personal and communal resources to address trauma and stress under extreme circumstances [13,14]. While some victims of IPV appeal to divine intervention, others take comfort in the social and emotional support provided by those around them to address the residual effects of violence [15,16,17]. Yet, some rely on their inner strength to cope with abuse since in some communitie, intimate partner violence is treated as a private matter. What is unclear, however, is whether groups within the Black population differ in coping practices and protective mechanisms that they employ, and the effect it may have on mental well-being. While sharing various similarities racially, African American and Caribbean Black women have unique histories, cultures, and experiences that may influence the methods used to address intimate victimization. This study addresses the mental health of US Black women and the role of coping and protective mechanisms on victimized women.

## 2. Background

### 2.1. IPV and Mental Health Outcomes

Previous research examining the outcomes of IPV among women revealed associations with multiple negative psychological symptoms [2,3,4]. PTSD, depression, and suicide are among the common sequelae identified in the research literature [14,18,19]. Although fewer studies have been conducted on US Black women, the mental health consequences resulting from IPV have largely mirrored that of the general population. A body of research has found that intimate partner violence places African American and Caribbean women at increased risk for a variety of mental health outcomes including substance abuse, anxiety, and mood disorders as well as suicide ideation and attempt [20,21].

### 2.2. Coping and Protective Mechanism of Mental Health

Despite the harm that IPV poses to health and well-being, scant investigations have focused on how some women cope or manage their IPV experiences to minimize these negative outcomes. Acts of opposition, the use of social and medical networks, religious activities, and even violence have been identified as behaviors used to combat IPV [22]. Some research has especially pointed to the strength and resilience of Black women in managing stressful and traumatic circumstances. Resilience can be viewed as a defense mechanism that enables people to thrive in the face of adversity [23]. Being resilient does not imply a symptom-free life, but rather the ability to meet adversity and move forward despite negative outcomes [24]. An examination of the association between resilience and mental health has found both a positive and negative relationship. In general, these studies suggest that resilience plays a role in helping individuals achieve a positive state of mental health and reducing negative indicators [25].

Spirituality and religiosity have also been identified as coping mechanisms for dealing with stress and trauma [26,27]. For some Black women facing adversity, the belief in a higher power, accessed through prayer, has been associated with positive health outcomes [28]. There is mounting evidence of the influence of spirituality and religiosity on health and well-being [29,30,31,32,33,34,35]. However, the literature has provided mixed outcomes as some studies have recognized spirituality and religiosity as protective factors of well-being, while such benefits were not found in other studies due to the negative emotions of guilt and shame it can impose on individuals [27,36,37].

Social support is a recognizable source of coping and protection that can benefit persons who face adverse circumstances [38,39,40]. Social support can come from various sources including family, friends, and institutional networks. The support can be both tangible and emotional in helping to safeguard individuals from unhealthy behaviors. Some scientific literature has linked social support to morbidity and mortality [41]. For example, research has found that perceived social support was associated with major depressive disorder among African American and Caribbean Blacks [42]. Additional research has found that emotional support from family members was inversely associated with depressive symptoms [38].

### 2.3. Moderators of IPV and Mental Health

Irrespective of the benefits of coping and protective mechanisms to health and well-being outlined, very few studies have addressed its effectiveness on Black women victims of intimate partner violence. Studies generally suggest that social support is a key factor in promoting good mental health among abused women through the provision of resources and the availability of networks to assist with the stress and the residual effect of violence [11,43,44]. For example, Kaslow et al. [45] earlier found that suicidal behavior was moderated by social support among victimized women. Coker and colleagues [46] also found that abused women with high social support were less likely to report anxiety, depression, PTSD symptoms, and suicide ideation than women reporting lower social support. Furthermore, social support moderated the association between IPV and mental health [46]. More recently, it was found that social support was related to better physical health, lower psychological distress, and lower incidence of depression among women experiencing IPV who use community services [47]. Among a racially diverse group of abused women, those who received low to moderate social support also had a greater likelihood of having depression compared to those who received higher levels of social support [48].

Although there has been very limited examination of spirituality, religiosity, and resilience as coping and protective mechanisms of mental health among victims of abuse, in a sample of low-income African American women it was found that symptoms of depression and anxiety were mediated by spiritual well-being [49]. Kaufman and colleagues [50] found that women who experience IPV had a lower level of mental health symptoms when high spirituality was present. Likewise, resilience was found to partially mediate the relationship between perceived stress and severe depression among Black women who were exposed to sexual violence.

## 3. Conceptual Framework

The Superwoman Schema (SWS) is a useful framework for examining Black women’s mental health outcomes as a consequence of IPV. Grounded in an understanding of Black women’s lives and the intersection of their race and gender identities, the SWS specifies several characteristics associated with Black womanhood: the obligation to show strength, to suppress emotions and to help others, resistance to vulnerability and dependency, and the power to achieve success even with limited resources [51,52,53]. Some Black women develop these characteristics and learn to take on the role of the strong Black woman as a cultural imperative. Although research on the SWS has revealed both benefits (preservation of self, family, and community) and liabilities (heightened health risks), a component of SWS may assist with understanding the responses of native and immigrant Black women facing IPV [54,55].

The Stress-Buffering Hypothesis also informs this research. The stress-buffering hypothesis purports that certain types of social support attenuate the negative effects of health outcomes associated with stress, and has been found to be helpful across the lifespan for individuals with physical and psychological conditions [56,57,58,59,60]. Essentially, the hypothesis focuses on the role that support mechanisms can play in the midst of stressful conditions that might be pathogenic to mental health [61].

### Study Rationale

Studies examining the mental health of US Black women have increased in recent years, but factors that enable some women to cope and maintain in the face of stress and adversity remains unclear. This research sought to add to our understanding by examining the coping and protective mechanisms of victimized US Black women. Using data from one of the most comprehensive studies of US Blacks we aim to: (1) address the association between severe intimate partner and the mental health risks among US Black women including African American and Caribbean Blacks; (2) explore the effect of coping mechanisms on the mental health of Black women, and how they might differ between groups; and (3) examine whether coping and protective mechanisms moderate the relationship between IPV and mental health conditions. It is unclear what effect these mechanisms might have on US Black women’s health disposition. We expected that moderators in the form of emotional family support, religiosity, spirituality, and resilience, would lessen the risk for poor mental health outcomes.

## 4. Materials and Methods

### 4.1. Sample

We analyze cross-sectional data from the National Survey of American Life (NSAL), a multistage probability sample of Blacks residing in the United States [62]. Collected between February 2001 and March 2003, the sample is inclusive of non-institutionalized adults. Structured interviews (in-person and phone) were administered to participants selected for the study. A total of 6082 participants completed interviews including: 890 non-Hispanic Whites; 3750 African American; and 1621 Caribbean Blacks. African Americans were those who identified as Black but without Caribbean roots. Participants self-identified as Caribbean Black if they were of African descent and were either: (a) of West Indian background, (b) from a Caribbean-area country, or (c) had parents or grandparents who were born in a Caribbean area county. Interviews, in general, were completed in 2 h and 20 min with a response rate of 72.3 percent. Participants received an honorarium of $50 for completing the survey. We specifically focus our analysis on the 3277 women of African descent collectively within the NSAL sample.

### 4.2. Mental Health Outcome Measures

Lifetime mood and anxiety disorders were assessed using a slightly modified version of the Diagnostic Statistical Manual of Mental Disorders fourth edition (DSM-IV) defined by the World Health Organization Composite International Diagnostic Interview (WHO CIDI). Mood disorder was inclusive of major depressive episode (MDE), dysthymia, major depressive disorder (MDD), and bipolar disorder (any). Anxiety disorders consisted of panic disorder, agoraphobia, generalized anxiety disorder (GAD), obsessive compulsive disorder (OCD), and posttraumatic stress disorder (PTSD). We further examined suicide ideation for this study. Suicide ideation was determined by the question, “Have you ever seriously thought about committing suicide?” Options of “yes” or “no” were provided to participants.

### 4.3. Moderator Measures

Religiosity and Spirituality. Individual questions were presented to participants regarding religiosity and spirituality. Within the literature, religiosity is referred to as an adherence to a set of organized beliefs and practices associated with a higher power; it may be practiced in the community or alone in private [31,63]. Spirituality refers to seeking inward meaning and purpose through inner experiences; it differs from religion in that it is generally free of rules and regulations associated with religious practices [31,63]. In this study, the questions posed to participants were: “How religious are you?” and “How spiritual are you?” The scales for these Likert measures ranged from very (1) to not at all (4).

Resilience. Resilience is the average of the eight items measured on a Likert scale ranging from (1) strongly agree to (4) strongly disagree. The items included were: (1) “There is really no way to solve some of the problems I have”; (2) “Sometimes I feel pushed around in life”; (3) “I have little control over the things that happens to me”; (4) “I can do anything I set my mind to”; (5) “I often feel helpless dealing with the problems of life”; (6) “What happens in the future mostly depends on me”; (7) “There is little I can do to change many of the important things in my life”; and (8) “I look to God for strength, support, and guidance.” They were reversed coded as appropriate. Collectively the internal consistency was 0.675. 

Emotional Family Support. Consisting of three items, the questions presented to participants were: (1) “How often family make you feel loved?”; (2) “How often family listen to your problems?”; and (3) “How often family express concern?” The response possibilities included (1) very often, (2) fairly often, (3) not too often, and (4) never. The internal consistency for this study was 0.776. The items were averaged for analysis.

### 4.4. Predictor Measures

Intimate Partner Violence. Severe physical intimate partner was addressed with the question, “Were you ever been badly beaten up by a spouse or romantic partner”? Binary response options (yes or no) were provided. To address the measure’s validity, we compared the NSAL IPV measure with the National Comorbidity Study Replication (NCS-R) dichotomously defined severe partner violence Conflict Tactic Scale (CTS) within the Collaborative Psychiatric Epidemiology Surveys (CPES). Two tests were conducted including the probability of agreement (OR = 4.5, CI = 1.49–14.98, *p* < 0.001) and area under the curve (AUC ≥ 0.6); they were found to have a fair association across approaches to estimating agreement [20].

### 4.5. Control Measures

The control variables were age (in years), marital status (married, partnered, separated or divorced, widowed, never married), employment status (employed, unemployed, not in the labor force), level of education (less than high school, high school graduate, some college, college), and poverty status (below and above). Poverty status is an income-to-poverty ratio measure consisting of participants’ household income divided by the 2001 US Census poverty threshold for the number of adults and children living in a household. Ratios below 1 indicate that participants’ household income is below the official poverty threshold; a ratio of 1.00 or greater indicates income above the poverty level [64].

### 4.6. Analytic Plan

The analyses were conducted using Stata 17.0 with a 0.05 alpha level to determine statistical significance. We conducted descriptive statistics and bivariate analysis (e.g., chi-square, *t*-test) to address sample distribution and differences. Multivariate logistic regression analyses were conducted across cohorts (i.e., Black, African American, Caribbean Black) to examine associations of mental health and severe intimate partner violence when moderated by coping mechanisms (e.g., family support × severe IPV). We specifically fit two models: one without and one with moderators. Due to the nature of the complex survey design, the sample was weighted and corrected for standard errors, clustering, stratification, and differential non-response [65].

## 5. Results

### Sample Characteristics

In general, the average age of sampled Black women was forty-three (m = 42.5) years (see Table 1). Over a third of women never married or were single (32.4%). The indicators of socio-economic status showed that almost 2 out 3 women were employed; the educational level within the population varied with more (36%) women completing a high school; and nearly three-quarters (71.6%) of women lived above the federal poverty threshold. A larger (52.3%) percentage of women in the sample were not too religious. Additionally, nearly half (47.4%) of the participants considered themselves not spiritual. Participants had a mean score of 3.4 on the measure of resilience. This was almost the same for the measure of emotional family support where the mean score was 3.3.

## 6. Multivariate Analysis Examining Mental Disorders and the Moderating Effect of Coping on the Relationship between IPV

### 6.1. Mood Disorder

Multivariate results show that severe intimate partner violence was associated with an increased risk for mood disorders (AOR = 2.38, *p* < 0.001) among US Black women controlling for other factors (see Table 2). Resilience moderated the relationship between IPV and mood order (AOR = 2.36, *p* < 0.001). With the absence of IPV, resilience was associated with reduced odds for mood disorders (AOR = 0.259, *p* < 0.001). The odds for mood disorder were further reduced (AOR = 0.967, *p* < 0.001) as US Black women aged. The opposite results were found for women who were separated or divorced where the odds (AOR = 1.49, *p* < 0.05) for mood disorders increased when compared to married women. College-educated women compared to high school educated women were also at increased odds (AOR = 1.93, *p* < 0.005) for mood disorders.

Subgroup analysis of African American women yielded similar results. To begin, severe IPV increased (AOR = 2.34, *p* < 0.001) the risk for mood disorder. Likewise, resilience moderated the relationship between IPV and mood disorder (AOR 2.48, *p* < 0.001). Even so, with the absence of IPV, resilience was typically associated with reduced odds (AOR = 0.239, *p* < 0.001) for mood disorder. The odds (AOR = 0.967, *p* < 0.001) for this condition were further reduced with age. The opposite was the case for college-educated women compared to women with a high school education where increasing odds (AOR = 1.78, *p* < 0.001) were found.

Among Caribbean Black women, severe intimate partner was associated with greater odds (AOR = 4.03, *p* < 0.01) for mood disorder. The relationship, however, was not moderated by other factors. Nonetheless, spirituality reduced the odds (AOR = 0.620, *p* < 0.05) for mood disorder. The odds (AOR = 0.971, *p* < 0.05) for mood disorder were further reduced with age. Conversely, there were increased odds for this condition among separated or divorced (AOR = 3.05, *p* < 0.05) and never married (single) (AOR = 3.01, *p* < 0.01) women when compared to married women. Caribbean women with a college (AOR = 5.64, *p* < 0.01) and some college (AOR = 4.15, *p* < 0.01) education were also at greater risk for mood disorder when compared with high school educated women.

### 6.2. Anxiety Disorder 

The analysis further showed an increased risk (AOR = 2.74, *p* < 0.001) for anxiety disorder among Black victims of severe IPV (see Table 3). Religiosity (AOR= 1.36, *p* < 0.05) also had a positive effect on anxiety. This was the opposite for resilience where lower odds (AOR= 0.319, *p* < 0.001) for this condition was found. Social factors contributed to anxiety disorder among US Black women. Such that, the odds (AOR = 0.977, *p* < 0.001) for anxiety reduced with age. However, increased odds (AOR = 1.64, *p* < 0.05) for this condition were found among widowed women compared to married women. 

As with the general sample, severe IPV increased the risk (AOR = 2.66, *p* < 0.001) for anxiety disorder among African American women. Likewise, religiosity increased the odds (AOR = 1.38, *p* < 0.05) for this disorder. The opposite was found when examining the effects of resilience on anxiety (AOR = 0.316, *p* < 0.001). Moreover, reduced odds (AOR = 0.977, *p* < 0.001) for anxiety were found with age.

Further analyses indicate that Caribbean Black women exposed to severe IPV were at increased risk (AOR= 4.94, *p* < 0.001) for anxiety disorder. The risk for anxiety disorder also increased for college-educated women (AOR= 2.45, *p* < 0.005) or women who had some college education (AOR = 3.30, *p* < 0.05) when compared to women with less than high school education. Living below the poverty threshold further increased the odds (AOR = 1.89, *p* < 0.05) for anxiety disorder when compared to those living above poverty. 

### 6.3. Suicide Ideation

Table 4 illustrate the association between severe IPV and the increased risk (AOR = 2.60, *p* < 0.001) for suicide ideation among US Black women. Specific moderators such as resilience reduced the odds (AOR = 0.498, *p* < 0.001) for suicide ideation. Likewise, emotional family support reduced the likelihood (AOR = 0.705, *p* < 0.01) of this behavior. Finally, the odds (AOR = 0.970, *p* < 0.001) for suicide ideation decreased with age. 

Subgroup analysis also showed that severe IPV increased (AOR = 2.52, *p* < 0.001) the risk for suicide ideation among African American women. Opposite to this, resiliency reduced the odds (AOR = 0.478, *p* < 0.001) for suicide ideation. This was true for family support where lower odds (AOR = 0.703, *p* < 0.001) were found. As African American women aged, the risks for suicide ideation (AOR = 0.971, *p* < 0.001) also decreased.

A slightly different pattern was found for Caribbean Black women. While severe IPV increased the odds (AOR = 5.83, *p* < 0.001) for suicide ideation among this subpopulation, specific coping and protection mechanisms were not associated with suicide ideation. Demographic factors, however, were associated with suicide ideation. For example, being college-educated increased the odds (AOR = 3.38, *p* < 0.05) for suicide ideation when compared to their less than high school educated counterparts. Opposite to these findings, women living below poverty compared to those living above the poverty threshold had lower odds (AOR =0.302, *p* < 0.05) for suicide ideation. The odds (AOR = 0.957, *p* < 0.001) for suicide ideation further reduced with age.

## 7. Discussion

Consistent with previous research, our study showed that IPV can be consequential to mental well-being [3,5,66]. Although we hypothesized that coping and protective mechanisms would lessen the potential for mental health conditions, this was not generally supported among women exposed to IPV. Our research found that resilience lost its protective effect when moderating the relationship between IPV and mood disorder. Specifically, resilience increased the risk for mood among victimized women. This may suggest that victimized Black women who exercise greater resilience might place themselves at risk for poor well-being. This was true for African American women, but not Caribbean women. While both African American women and Black Caribbean women embrace aspects of the Superwomen Syndrome (SWS), its use by Black Caribbean women increase their ability to tolerate distress [67]. Graham & Clarke [68] also noted that Caribbean women may access a broader range of strategies to manage distress. Further highlighted was the idea that their cultural notions of strength were linked to generations of African-heritage women who have overcome multiple forms of adversity. This multi-pronged approach to coping may partially explain the differences in resilience and well-being outcomes between African American and Caribbean Black women.

Our study further showed that in the absence of IPV, there was a protective effect of certain mechanisms that contributed to the reduction of certain mental health conditions among US Black women. Surprisingly, religiosity increased the likelihood of anxiety disorder among this population. The idea that religion imparts a calming effect on individuals was not supported. Instead, it can be assumed that being religious might result in some individuals ignoring formal care thus worsening women’s mental well-being. Furthermore, some churches may work to discourage women from secular help-seeking which may exacerbate the problem [36,37]. Interestingly enough, this relationship was found for African American women but not for Caribbean Black women. Instead, spirituality had the opposite effect on mood disorder in Caribbean women. Spirituality was associated with a lower likelihood of mood disorder among Caribbean women which was not the case for African American women.

While this divergent finding was unanticipated, a partial explanation may lie in the fact that African American women might adhere to more formal religious observances than Caribbean women, who may share stronger ties to syncretic or blended African and western religions practices [69,70]. This cultural difference in religion may explain why the opposite was true for spirituality, practices which is more closely associated with diverse African religious and spiritual traditions. Personal and practical rewards often accompany traditional religious practices, whereas spirituality is a more individualized experience associated with higher levels of inner personal growth [27,31,36,63]. Even so, this study demonstrated that resilience reduced the likelihood of mood and anxiety across groups; a finding that would align with positive aspects of SWS, which include preservation of self [52,53].

There were other noted differences and similarities between African American and Caribbean Blacks particularly as it relates to suicide ideation. For example, emotional family support reduced the likelihood of suicide ideation among African American women. This was also evident for resilience among this population and could be aligned with the stress-buffering hypothesis. In both instances, the associations did not hold for Caribbean women. One plausible explanation is that African American women are likely to have a broader network of family support available to them by virtue of being indigenous to America; larger social networks have been associated with greater resilience [71,72,73]. Caribbean women may not have the same breadth and depth of positive family support immediately accessible, and this is especially important when considering the dire ramifications of persistent suicidal ideation [4,72].

A consistent finding across the populations was that age contributed to the reduced risk of mental health conditions and behaviors. Such a relationship may be a result of having more experience in coping with life trauma, and more openness to utilizing a range of resources, factors which accrue across the lifespan [74]. Other demographic characteristics were also associated with both positive and negative well-being among Black women in the sample. For example, among US Black and Caribbean women, having a college education contributed negatively to specific mental disorders and behaviors, with a more pronounced effect noted for Caribbean women. This finding is difficult to explain, especially since education is typically associated with positive well-being. However, it has been recognized as a confounding factor in other research [24,47,74,75]. A relatively positive mental health status has been noted in recent studies of other minority and immigrant populations who encounter crises [76,77,78]. As the number of Black women pursuing college degrees continues to increase, the need for targeted research to explain the impact of education on the mental health sequelae of Black women enduring IPV is crucial.

Poverty also contributed both positively and negatively to specific mental disorders and behaviors among Caribbean women, which highlights the need for further investigation of how social circumstances may impact mental health outcomes [66]. Similarly, the association between relationship status and mood disorders in US Caribbean women warrants further investigation, as findings indicated that being single, separated, or divorced increases the likelihood of mood disorder. Given that psychological well-being in humans is complex, employing qualitative methodologies in future studies may be helpful to understanding the psychological well-being of women facing crises and the influence of socio-demographic factors [79,80,81].

### Limitations and Strength of Study

It is important to consider a few shortcomings when interpreting the study findings including the use of data that are more than a decade old. Despite the age of the data, this study examined associations that are not likely to change during that period. Along this line, causal inferences cannot be drawn due to the cross-sectional nature of the data. It should be cautioned that some scales (i.e., resilience) utilized in this study did not yield the highest internal consistency. Third, there were other coping and protective mechanisms not explored, which women within that population traditionally turn to (i.e., friends, cultural factors) to address trauma or abuse. Finally, the focus of this study was only on physical intimate partner violence. This could not be avoided as other measures of violence (i.e., psychological, sexual) known to negatively affect the health of women were not present in the dataset.

Irrespective of the shortcomings highlighted, this study validates and draws our attention to potential intervention and preventative measures that have traditionally been used by Black women to cope with adversities. Additionally, this study examined multiple protective and coping mechanisms that are culturally appropriate for the populations under examination. Furthermore, comparisons were made between two ethnic groups within the US Black populations to understand the effectiveness of the protective and coping mechanism on women’s mental outcome, which to our knowledge, has not been explored thoroughly using representative data, especially among a population that is vulnerable to intimate partner victimization.

## 8. Conclusions

This research suggests that US Black women exposed to intimate victimization and other trauma are susceptible to a host of negative outcomes that could be moderated by other factors. These factors have often been discussed in the literature, but rarely explored using population-based data. Our study provides support for resiliency, spirituality, and social support as an effective coping and protective mechanism that can help to preserve the mental health of US Black women. However, there are certain protective and coping strategies that might have differing effects on victimized women from different segments of the population. Although our study provides some evidence that the mechanisms explored provided some protection from mental health disorders and behavior, it should also be cautioned that other coping and protective mechanisms might exacerbate certain conditions. Even though it is evident that these coping and protective factors can be useful intervention and prevention measures, they should be culturally tailored specifically to respective populations. Given this point, an important takeaway of this study is that counselors and clinicians should consider exploring some of the coping mechanisms examined in this study (and others) as viable treatment sources to assist Black women who have been exposed to intimate victimization and other adversities.

## Figures and Tables

**Table 1:** Sample Characteristics.

Characteristics	US Black Women	African American	Caribbean Black	Ethnic Difference Statistics F-Test Statistics
**Age**	42.5	42.6	40.5	0.46
**Marital Status**				10.86 ***
Married	27.4	27.3	28.5	
Partnered	8.4	8.2	11.6	
Sep-Div	20.3	20.2	21.4	
Widowed	11.5	11.8	6.4	
Never Married	32.4	32.4	32.1	
**Employment Status**				6.82 **
Employed	63.7	63.2	71.1	
Unemployed	11.1	11.1	10.3	
Not in labor force	25.2	25.7	18.7	
**Education**				5.74 **
Less than HS	24.8	25.1	20.3	
HS graduate	36.0	36.3	30.5	
Some college	24.8	24.5	29.0	
College	14.4	14.1	20.3	
**Poverty**				14.95 ***
Above	71.6	71.0	80.3	
Below	28.4	29.0	19.7	
**Religiosity**				6.63 ***
Not religious	35.6	36.2	27.0	
Not too religious	52.3	52.1	56.5	
Fairly religious	9.5	9.2	14.6	
Very Religious	2.5	2.6	2.0	
**Spirituality**				3.57 *
Not spiritual	47.4	47.9	40.2	
Not too spiritual	44.2	43.9	48.8	
Fairly spiritual	7.5	7.5	7.8	
Very spiritual	1.0	0.9	3.3	
**Resiliency**	3.4	3.4	3.4	0.03
**Emotional Family Support**	3.3	3.3	3.3	0.35

Note: Percentage are weighted; * *p* < 0.05; ** *p* < 0.01; *** *p* < 0.001.

**Table 2:** Multivariate Analysis Examining IPV and Moderators on Mood Disorder.

Characteristics	US Black Women AOR (CI)		African American AOR(CI)		Caribbean Black AOR (CI)	
	Block 1 No Moderators	Block 2 Moderators	Block 1 No Moderators	Block 2 Moderators	Block 1 No Moderators	Block 2 Moderators
**Age**	0.967 (0.956–0.979) ***	0.967 (0.955–0.978) ***	0.967 (0.954–0.980) ***	0.967 (0.954–0.979) ***	0.971 (0.947–0.995) *	0.971 (0.947–0.995) *
**Marital Status**						
Married	1	1	1	1	1	1
Partnered	1.21 (0.657–2.24)	1.28 (0.693–2.38)	1.18 (0.609–2.29)	1.25 (0.643–2.44)	1.94 (0.486–7.75)	2.06 (0.500–8.50)
Sep-Div	1.52 (1.05–2.20) *	1.49 (1.03–2.15) *	1.44 (0.969–2.15)	1.41 (0.955–2.10)	3.15 (0.999–9.96) *	3.05 (0.977–9.54) *
Widowed	1.50 (0.719–3.11)	1.44 (0.700–2.95)	1.48 (0.689–3.18)	1.42 (0.669–3.00)	0.939 (0.124–7.09)	0.880 (0.120–6.47)
Never Married	0.914 (0.586–1.43)	0.928 (0.596–1.45)	0.837 (0.517–1.35)	0.855 (0.530–1.38)	2.95 (1.50–5.82) ***	3.01 (1.58–5.72) **
**Employment Status**						
Employed	1	1	1	1	1	1
Unemployed	0.791 (0.512–1.22)	0.763 (0.488–1.19)	0.802 (0.505–1.28)	0.767 (0.476–1.24)	0.653 (0.260–1.64)	0.628 (0.229–1.72)
Not in labor force	0.832 (0.546–1.27)	0.867 (0.576–1.31)	0.782 (0.494–1.24)	0.815 (0.523–1.27)	1.38 (0.686–2.79)	1.49 (0.684–3.24)
**Education**						
Less than high school	1	1	1	1	1	1
HS graduate	0.946 (0.646–1.38)	0.925 (0.639–1.34)	0.935 (0.623–1.40)	0.915 (0.617–1.36)	1.22 (0.346–4.27)	1.38 (0.403–4.73)
Some college	1.46 (0.948–2.26)	1.44 (0.920–2.25)	1.37 (0.854–2.18)	1.34 (0.828–2.17)	3.61 (1.49–8.72) **	4.15 (1.62–10.61) **
College	1.91 (1.16–3.14)	1.93 (1.19–3.15) **	1.77 (1.04–3.00) *	1.78 (1.06–3.00) *	5.07 (1.51–16.98) **	5.64 (1.58–20.13) **
**Poverty**						
Above	1	1	1	1	1	1
Below	1.10 (0.795–1.51)	1.10 (0.803–1.52)	1.06 (0.750–1.51)	1.07 (0.758–1.51)	1.72 (0.796–3.70)	1.80 (0.897–3.60)
**IPV**						
No	1	1	1	1	1	1
Yes	2.38 (1.75–3.23) ***	2.73 (2.01–3.71) ***	2.34 (1.69–3.23) ***	2.74 (1.99–3.77) ***	4.03 (1.53–10.65) **	3.37 (1.50–7.55) **
**Religiosity**	1.05 (0.845–1.30)	0.986 (0.767–1.27)	1.05 (0.828–1.32)	0.986 (0.751–1.29)	1.02 (0.722–1.45)	0.926 (0.648–1.32)
**Spirituality**	0.899 (0.747–1.08)	0.849 (0.700–1.03)	0.904 (0.737–1.11)	0.852 (0.687–1.06)	0.707 (0.456–1.10)	0.620 (0.394–0.975) *
**Resilience**	0.324 (0.254–0.413) ***	0.259 (0.198–0.340) ***	0.305 (0.236–0.393) ***	0.239 (0.179–0.318) ***	0.642 (0.371–1.11)	0.614 (0.329–1.14)
**Family Support**	0.918 (0.740–1.14)	0.859 (0.657–1.12)	0.909 (0.719–1.15)	0.840 (0.629–1.12)	1.02 (0.673–1.56)	1.20 (0.685–2.10)
**IPV × Religiosity**						
No		1		1		1
Yes		1.22 (0.696–2.15)		1.21 (0.666–2.20)		2.13 (0.757–5.98)
**IPV × Spirituality**						
No		1		1		1
Yes		1.18 (0.712–1.96)		1.16 (0.678–2.00)		1.52 (0.587–3.95)
**IPV × Resiliency**						
No		1		1		1
Yes		2.36 (1.22–4.56) **		2.48 (1.23–4.99) **		1.55 (0.353–6.81)
**IPV × Family Support**						
No		1		1		1
Yes		1.26 (0.827–1.92)		1.31 (0.832–2.06)		0.579 (0.288–1.16)

Note: * *p* < 0.05; ** *p* < 0.01; *** *p* < 0.001.

**Table 3:** Multivariate Analysis Examining IPV and Moderators on Anxiety.

Characteristics	US Black Women AOR (CI)		African American AOR(CI)		Caribbean Black AOR (CI)	
	Block 1 No Moderators	Block 2 Moderators	Block 1 No Moderators	Block 2 Moderators	Block 1 No Moderators	Block 2 Moderators
**Age**	0.967 (0.966–0.988) ***	0.977 (0.967–0.988) ***	0.977 (0.966–0.989) ***	0.977 (0.966–0.989) ***	0.977 (0.956–0.998) *	0.975 (0.955–0.995) *
**Marital Status**						
Married	1	1	1	1	1	1
Partnered	1.26 (0.723–2.20)	1.27 (0.725–2.24)	1.28 (0.710–2.31)	1.29 (0.711–2.35)	0.985 (0.239–4.05)	0.960 (0.233–3.96)
Sep-Div	1.29 (0.876–1.89)	1.24 (0.848–1.82)	1.30 (0.865–1.96)	1.26 (0.837–1.89)	0.852 (0.298–2.43)	0.793 (0.262–2.40)
Widowed	1.68 (1.05–2.69) *	1.64 (1.01–2.66) *	1.68 (1.03–2.74) *	1.64 (0.986–2.71)	0.866 (0.184–4.07)	0.883 (0.210–3.71)
Never Married	0.928 (0.550–1.57)	0.940 (0.555–1.59)	0.883 (0.504–1.55)	0.898 (0.511–1.58)	1.98 (0.775–5.07)	1.86 (0.697–4.96)
**Employment Status**						
Employed	1	1	1	1	1	1
Unemployed	0.953 (0.690–1.31)	0.942 (0.683–1.30)	0.973 (0.696–1.36)	0.961 (0.690–1.34)	0.685 (0.179–2.63)	0.717 (0.181–2.84)
Not in the labor force	0.998 (0.655–1.52)	1.02 (0.675–1.55)	0.967 (0.613–1.52)	0.991 (0.630–1.56)	1.35 (0.769–2.39)	1.40 (0.764–2.57)
**Education**						
Less than high school	1	1	1	1	1	1
HS graduate	0.780 (0.562–1.08)	0.772 (0.559–1.07)	0.745 (0.526–1.05)	0.739 (0.525–1.04)	2.81 (0.868–9.08)	2.71 (0.878–8.40)
Some college	0.911 (0.602–1.38)	0.909 (0.600–1.38)	0.860 (0.552–1.34)	0.861 (0.552–1.34)	3.59 (1.41–9.10) **	3.30 (1.26–8.68) *
College	0.899 (0.550–1.47)	0.911 (0.559–1.48)	0.878 (0.521–1.48)	0.891 (0.532–1.49)	2.49 (1.06–5.80) *	2.45 (1.07–5.60) *
**Poverty**						
Above	1	1	1	1	1	1
Below	1.05 (0.828–1.34)	1.05 (0.827–1.33)	1.02 (0.790–1.32)	1.01 (0.787–1.31)	1.87 (0.995–3.52) *	1.89 (0.990–3.61) *
**IPV**						
No	1	1		1		1
Yes	2.74 (2.07–3.62) ***	3.04 (2.33–3.96) ***	2.66 (1.98–3.56) ***	2.94 (2.23–3.89) ***	4.94 (2.66–9.18) ***	5.56 (2.78–11.19) ***
**Religiosity**	1.26 (1.01–1.56) *	1.36 (1.04–1.77) *	1.26 (1.01–1.58) *	1.38 (1.04–1.82) *	1.19 (0.723–1.95)	1.11 (0.614–2.00)
**Spirituality**	0.843 (0.663–1.07)	0.791 (0.602–1.04)	0.847 (0.655–1.10)	0.790 (0.586–1.07)	0.836 (0.544–1.28)	0.840 (0.541–1.30)
**Resilience**	0.371 (0.291–0.475) ***	0.319 (0.227–0.448) ***	0.370 (0.285–0.479) ***	0.316 (0.219–0.455) ***	0.286 (0.119–0.689) **	0.278 (0.103–0.746) *
**Family Support**	0.939 (0.785–1.12)	0.899 (0.720–1.12)	0.939 (0.776–1.14)	0.902 (0.710–1.15)	1.03 (0.579–1.85)	0.983 (0.508–1.90)
**IPV × Religiosity**						
No		1		1		1
Yes		0.745 (0.440–1.26)		0.723 (0.415–1.26)		1.76 (0.440–7.07)
**IPV × Spirituality**						
No		1		1		1
Yes		1.24 (0.777–1.97)		1.25 (0.758–2.08)		0.942 (0.341–2.60)
**IPV × Resiliency**						
No		1		1		1
Yes		1.81 (0.933–3.52)		1.83 (0.900–3.71)		1.44 (0.416–4.99)
**IPV × Family Support**						
No		1		1		1
Yes		1.17 (0.890–1.54)		1.16 (0.866–1.54)		1.34 (0.525–3.44)

Note: * *p* < 0.05; ** *p* < 0.01; *** *p* < 0.001.

**Table 4:** Multivariate Analysis Examining IPV and Moderators on Suicide Ideation.

Characteristics	US Black Women AOR (CI)		African American AOR(CI)		Caribbean Black AOR (CI)	
	Block 1 No Moderators	Block 2 Moderators	Block 1 No Moderators	Block 2 Moderators	Block 1 No Moderators	Block 2 Moderators
**Age**	0.970 (0.956–0.984) ***	0.970 (0.956–0.984) ***	0.971 (0.956–0.986) ***	0.971 (0.956–0.986) ***	0.956 (0.933–0.980) ***	0.957 (0.933–0.981) ***
**Marital Status**						
Married	1	1	1	1	1	1
Partnered	1.24 (0.636–2.43)	1.28 (0.664–2.48)	1.30 (0.632–2.68)	1.35 (0.661–2.75)	0.502 (0.139–1.82)	0.504 (0.128–1.98)
Sep-Div	0.962 (0.611–1.51)	0.945 (0.602–1.49)	0.980 (0.605–1.59)	0.963 (0.595–1.56)	0.276 (0.068–1.12)	0.306 (0.078–1.20)
Widowed	0.845 (0.412–1.73)	0.827 (0.408–1.68)	0.840 (0.392–1.80)	0.821 (0.387–1.74)	0.602 (0.071–5.10)	0.604 (0.067–5.41)
Never Married	0.913 (0.528–1.58)	0.921 (0.533–1.59)	0.857 (0.476–1.54)	0.871 (0.482–1.58)	1.45 (0.811–2.59)	1.54 (0.835–2.84)
**Employment Status**						
Employed	1	1	1	1	1	1
Unemployed	1.20 (0.775–1.85)	1.16 (0.748–1.81)	1.25 (0.782–1.98)	1.20 (0.749–1.94)	0.400 (0.115–1.39)	0.333 (0.071–1.56)
Not in the labor force	0.956 (0.652–1.40)	0.979 (0.662–1.45)	0.909 (0.604–1.37)	0.934 (0.616–1.42)	1.97 (0.519–7.44)	2.06 (0.557–7.64)
**Education**						
Less than high school	1	1	1	1	1	1
HS graduate	0.926 (0.619–1.39)	0.913 (0.613–1.36)	0.922 (0.603–1.41)	0.911 (0.597–1.39)	1.72 (0.622–4.77)	2.32 (0.770–7.02)
Some college	0.967 (0.601–1.56)	0.960 (0.586–1.57)	0.973 (0.586–1.62)	0.969 (0.569–1.65)	1.06 (0.368–3.08)	1.49 (0.474–4.68)
College	0.624 (0.336–1.16)	0.623 (0.337–1.15)	0.547 (0.275–1.09)	0.547 (0.277–1.08)	2.68 (0.904–7.94)	3.38 (0.988–11.59) *
**Poverty**						
Above	1	1	1	1	1	1
Below	1.08 (0.747–1.55)	1.08 (0.758–1.54)	1.13 (0.766–1.66)	1.13 (0.779–1.65)	0.343 (0.122–0.961) *	0.302 (0.113–0.805) *
**IPV**						
No	1	1		1	1	1
Yes	2.60 (1.79–3.77) ***	2.85 (2.01–4.03) ***	2.52 (1.70–3.75) ***	2.80 (1.95–4.01) ***	5.83 (2.62–12.98) ***	4.09 (1.69–9.91) ***
**Religiosity**	0.990 (0.697–1.41)	0.943 (0.614–1.45)	1.01 (0.689–1.47)	0.959 (0.603–1.53)	0.828 (0.606–1.13)	0.749 (0.499–1.12)
**Spirituality**	0.840 (0.613–1.15)	0.823 (0.576–1.18)	0.823 (0.584–1.16)	0.800 (0.540–1.18)	1.14 (0.677–1.92)	1.13 (0.646–1.99)
**Resilience**	0.552 (0.418–0.728) ***	0.498 (0.345–0.718) ***	0.541 (0.406–0.721) ***	0.478 (0.326–0.702) ***	0.711 (0.337–1.50)	0.770 (0.335–1.77)
**Family Support**	0.773 (0.650–0.918) **	0.705 (0.546–0.910) **	0.779 (0.649–0.934) **	0.703 (0.535–0.925) **	0.622 (0.392–0.985) *	0.712 (0.422–1.20)
**IPV × Religiosity**						
No		1		1		1
Yes		1.15 (0.559–2.36)		1.13 (0.522–2.44)		1.34 (0.421–4.28)
**IPV × Spirituality**						
No		1		1		1
Yes		1.03 (0.550–1.93)		1.04 (0.529–2.06)		0.783 (0.306–2.00)
**IPV × Resiliency**						
No		1		1		1
Yes		1.45 (0.772–2.74)		1.54 (0.791–3.00)		0.434 (0.077–2.45)
**IPV × Family Support**						
No		1		1		1
Yes		1.34 (0.779–2.31)		1.39 (0.765–2.52)		0.495 (0.210–1.17)

Note: * *p* < 0.05; ** *p* < 0.01; *** *p* < 0.001.

## Data Availability

The public use NSAL data is archived at the University of Michigan-ICPSR.
